# Genetic and physiological analysis of tolerance to acute iron toxicity in rice

**DOI:** 10.1186/s12284-014-0008-3

**Published:** 2014-05-30

**Authors:** Lin-Bo Wu, Mohamad Yusser Shhadi, Glenn Gregorio, Elsa Matthus, Mathias Becker, Michael Frei

**Affiliations:** 1 Plant Nutrition, Institute for Crop Science and Resource Conservation (INRES), University of Bonn, Karlrobert-Kreiten-Straße 13, Bonn, 53115, Germany; 2 Plant Breeding, Genetics and Biotechnology Division, International Rice Research Institute, Los Baños, Philippines

**Keywords:** Iron toxicity, Oryza sativa L., Quantitative trait locus, Reactive oxygen species, Tolerance mechanism

## Abstract

**Background:**

Fe toxicity occurs in lowland rice production due to excess ferrous iron (Fe^2+^) formation in reduced soils. To contribute to the breeding for tolerance to Fe toxicity in rice, we determined quantitative trait loci (QTL) by screening two different bi-parental mapping populations under iron pulse stresses (1,000 mg L^−1^ = 17.9 mM Fe^2+^ for 5 days) in hydroponic solution, followed by experiments with selected lines to determine whether QTLs were associated with iron exclusion (*i.e*. root based mechanisms), or iron inclusion (*i.e*. shoot-based mechanisms).

**Results:**

In an IR29/Pokkali F_8_ recombinant inbred population, 7 QTLs were detected for leaf bronzing score on chromosome 1, 2, 4, 7 and 12, respectively, individually explaining 9.2-18.7% of the phenotypic variation. Two tolerant recombinant inbred lines carrying putative QTLs were selected for further experiments. Based on Fe uptake into the shoot, the dominant tolerance mechanism of the tolerant line FL510 was determined to be exclusion with its root architecture being conducive to air transport and thus the ability to oxidize Fe^2+^ in rhizosphere. In line FL483, the iron tolerance was related mainly to shoot-based mechanisms (tolerant inclusion mechanism). In a Nipponbare/Kasalath/Nipponbare backcross inbred population, 3 QTLs were mapped on chromosomes 1, 3 and 8, respectively. These QTLs explained 11.6-18.6% of the total phenotypic variation. The effect of QTLs on chromosome 1 and 3 were confirmed by using chromosome segment substitution lines (SL), carrying Kasalath introgressions in the genetic background on Nipponbare. The Fe uptake in shoots of substitution lines suggests that the effect of the QTL on chromosome 1 was associated with shoot tolerance while the QTL on chromosome 3 was associated with iron exclusion.

**Conclusion:**

Tolerance of certain genotypes were classified into shoot- and root- based mechanisms. Comparing our findings with previously reported QTLs for iron toxicity tolerance, we identified co-localization for some QTLs in both pluse and chronic stresses, especially on chromosome 1.

## Background

Fe is an essential element in plants that is involved in many physiological processes, but that can also be toxic when provided in excess. In well-aerated soils, Fe is present as ferric hydroxides with low plant availability (Conte and Walker [2011]). However, in anaerobic soils and at low redox potential (Eh), Fe is reduced to its soluble form Fe^2+^ and can be taken up excessively by plants. In plant tissues, Fe^2+^ participates in Fenton reactions, catalyzing the generation of hydroxyl radicals (·OH) and other reactive oxygen species (ROS) (Briat and Lebrun [1999]; Thongbai and Goodman [2000]). These radicals cause irreversible damage to membrane lipids, proteins and nucleic acids (Becana et al. [1998]). Eventually they oxidize chlorophyll and subsequently reduce leaf photosynthesis (Pereira et al. [2013]), thereby leading to yield reductions. The typical symptoms associated with iron toxicity are leaf discoloration (bronzing) and reddish spots (Ponnamperuma et al. [1955]). Yield losses associated with iron toxicity commonly range from 15% to 30%. However, in the case of severe toxicity at younger stage, complete crop failure can occur (Audebert and Sahrawat [2000]).

In the field, several types of Fe toxic conditions occur, differing by landscape and soil type attributes, the Fe concentrations in the solutions, and the physiological stage at which the stress occurs. Three types of Fe toxic conditions were proposed (Becker and Asch [2005]). (i) Due to high content of Fe^2+^ in acid sulfate soils, toxicity symptoms on plants can be observed during the whole growth period. Yield losses range from 40% to 100%. (ii) In acid clay soils, high Fe concentrations typically occur at around one month or more after transplanting. Leaf bronzing symptoms appear mainly during the late vegetative growth stage while genotypes are transplanted in the dry season with high vapor pressure deficits. (iii) In inland valleys, interflow water containing large amounts of Fe^2+^ can be flushed at the onset of the rainy season from adjacent slopes formed on highly weathered soils into the poorly drained valley bottoms with often sandy soils with low cation exchange capacity and low available P. Symptoms can be observed at the early development stages. Yield losses can range from 30-70%, but when severe toxicity occurs at the seedling stage, total crop failure can occur. While various soil, water and nutrient management strategies have been suggested to counteract negative effects of excess Fe in solution, the most promising approach is to use tolerant genotypes.

To adapt rice plants to these varying Fe toxic conditions, three types of tolerance mechanisms have been proposed. Type I refers to exclusion of Fe^2+^ at the root level. Root oxidizing power due to oxygen release or enzymatic oxidation is responsible for the oxidization and precipitation of Fe^2+^ on the root surface, thus avoiding excess Fe^2+^ from uptake into rice shoots (Ando et al. [1983]; Green and Etherington [1977]). Type II refers to the inclusion but subsequent avoidance of Fe^2+^*via* internal distribution and storage in a less reactive form. Thus, ferritin is a promising candidate protein as it can accommodate up to 4,000 Fe atoms in a safe and bio-available form (Briat et al. [2010]). Type III refers to inclusion and tolerance to ROS formed in the Fenton reactions. Anti-oxidants such as ascorbic acid, and reduced glutathione can scavenge ROS (Fang et al. [2001]; Gallie [2013]), and antioxidant enzymes, such as superoxide dismutase, peroxidase and catalase reportedly protect plants from ROS damage (Bode et al. [1995]; Fang and Kao [2000]; Fang et al. [2001]).

A number of screening and mapping experiments for tolerance to Fe toxicity in rice have reported somewhat contradictory tolerance rankings. For example, the *japonica* variety, Azucena was screened in 250 mg L^−1^ Fe^2+^ for 4 weeks in hydroponics and classified as tolerant (Wu et al. [1997]; Dufey et al. [2009]). However, it showed high susceptibility under a pulse stress of 1,500 mg L^−1^ Fe^2+^ (Engel et al. [2012]). Another two varieties, WITA 1 and Matkandu from Africa Rice Center and Malaysia, respectively, were moderately tolerant in Fe-toxic soils in Korhogo, Ivory Coast, but responded sensitively to Fe stress in Kilissi, Guinea (Audebert and Fofana [2009]). Yet another *indica* genotype, Pokkali was screened to be sensitive on an acid sulfate soil with chronic toxicity in the Philippines (Gregorio et al. [2002]), but it showed marked tolerance when treated with an Fe pulse stress of 1,500 mg L^−1^ Fe^2+^ in hydroponics (Engel et al. [2012]). These examples illustrate contradictory performances of the same genotypes in different screening studies. Due to the diversity of conditions under which Fe toxicity occurs and the different stress types and intensities, different crop adaptive strategies are required. To overcome the limitations posed by contradictory tolerance rankings, a deeper understanding of the physiological mechanisms of adaptation to different Fe toxic conditions and the genetic factors behind those mechanisms is required.

The mapping of QTL is an effective way to dissect genetic factors underlying phenotypic traits such as Fe stress tolerance. Several previous studies have reported QTLs for tolerance in diverse Fe toxic conditions (Dufey et al. [2009]; Dufey et al. [2012]; Fukuda et al. [2012]; Shimizu [2009]; Wan et al. [2003a]; Wan et al. [2003b]; Wan et al. [2005]; Wu et al. [1997]; Wu et al. [1998]) but the physiological tolerance mechanisms behind these QTLs have not been specified and remain still unclear. Our aim was therefore to dissect genetic factors associated with tolerance to a specific type of Fe toxicity by mapping of QTL, and to classify the physiological tolerance mechanism underlying these QTLs. We focused on intensive pulse stresses at the vegetative stage, as they typically occur during rainfall events in inland valleys (Audebert and Sahrawat [2000]). We first screened the parents of two mapping populations segregating in tolerance to Fe pulse stress, subsequently conducted two QTL mapping experiments with these populations, and finally investigated the physiological mechanisms underlying tolerance using selected lines from the mapping populations.

## Results

### The screening of parental lines

**Figure 1:**
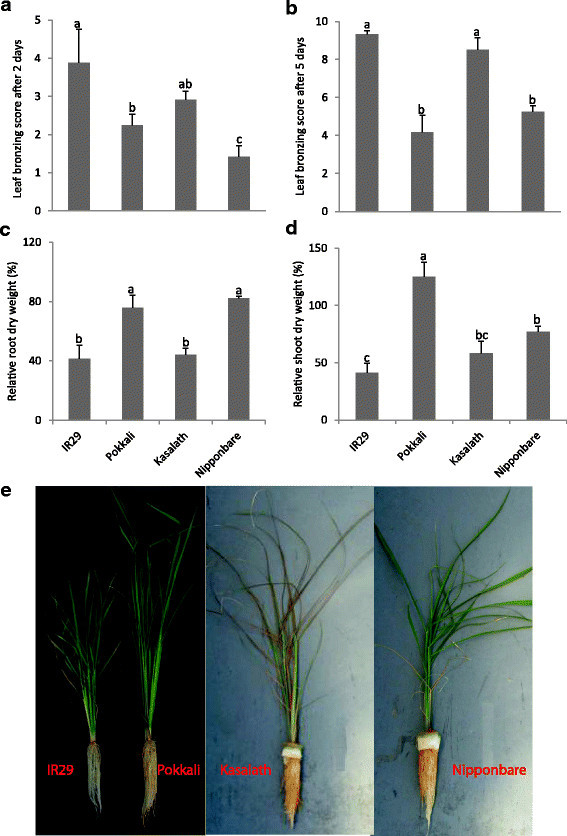
**Parents of two mapping populations were screened after a pulse stress of 1,000 mg L**^**−1**^**Fe**^**2+**^**for 5 days. (a)** Leaf bronzing score after 2-days-treatment, **(b)** leaf bronzing score after 5-days-treatment, **(c)** relative root dry weight after 5-days-treatment and **(d)** relative shoot dry weight. **(e)** Photos of partental lines after 5-days-treatment were determined. Bars represent standard errors of the mean (n = 6). Different letters above data points indicate significant differences between genotypes by LSD-test (*p* < 0.05).

The parental lines of two mapping populations were exposed to a pulse stress of 1,000 mg L^−1^ Fe^2+^ in hydroponics. The genotypes IR29 and Pokkali showed a significant difference (*p* < 0.01) in leaf bronzing score after 2 and 5 days of treatments (Figure 1a, b). The relative root and shoot dry weights of Pokkali were significantly higher than those of IR29 (Figure 1c, d). Also the bronzing scores after 2 and 5 days of stress exposure and the root biomass of Nipponbare were significantly lower than of those of Kasalath (Figure 1a, b), but no significant difference was found in shoot biomass (Figure 1d). Pokkali showed markedly higher tolerance than IR29 in terms of symptom score and relative shoot and root growth. Nipponbare was more tolerant than Kasalath in terms of symptom score and root growth. Therefore, the populations derived from these parents were considered suitable for QTL analysis.

### QTL analysis in IR29/Pokkali population

In the IR29/Pokkali population, significant QTLs were only detected for the trait leaf bronzing score after 5 days of stress exposure. A total of 7 QTLs were mapped on chromosome 1, 2, 4, 7, and 12, respectively (Table 1). Standard nomenclature for QTLs was adopted (McCouch et al. [1997]). On chromosome 1, two putative QTLs were detected: *q*FETOX-1-1 was located at the position of 12.9 Mb explaining 10.6% of the phenotypic variation. Another QTL, *q*FETOX-1-2 was highly associated with two linked markers at the position of 36.8-38.2 Mb. It explained 12.7-16% of the phenotypic variation. On chromosome 2, *q*FETOX-2 was found at 31.2 Mb, which explained 10.3% of the phenotypic variation. *q*FETOX-4-1 covering four linked markers was mapped at 7.4-12.0 Mb on chromosome 4. At the position of 20 Mb on chromosome 4, *q*FETOX-4-2 which explained 9.9% of the phenotypic variation was detected. Another two QTLs, *q*FETOX-7 and *q*FETOX-12 were mapped at 3.7 Mb on chromosome 7 and 27.6 Mb on chromosome 12, respectively. Except for *q*FETOX-1-2, the tolerance alleles of all QTLs were provided by Pokkali.

**Table 1:** **QTLs associated with leaf bronzing scores after 5 days of Fe**^**2+**^**stress exposure in IR29/Pokkali population by single marker regression**

**QTL**^**a**^	**Chromosome**	**Associated marker**^**b**^	**Position**^**c**^**(Mb)**	**-log** ***p*** **(F)**^**d**^	**Additive effect**^**e**^	**R**^**2**^**(%)**^**f**^	**Tolerance allele**^**g**^
*q*FETPX-1-1	1	id1008684	12.9	3.57	0.82	10.6	Pokkali
*q*FETOX-1-2	1	id1021920	36.8	4.23	−0.72	12.7	IR29
	1	id1023158	38.2	5.25	−0.81	16.0	
*q*FETOX-2	2	id2013434	31.2	3.47	0.94	10.3	Pokkali
*q*FETOX-4-1	4	id4002852	7.4	6.12	0.85	18.7	Pokkali
	4	id4002913	8.1	6.14	0.86	18.7	
	4	id4003259	10.0	5.95	0.84	18.1	
	4	id4003727	12.0	3.15	0.82	9.2	
*q*FETOX-4-2	4	id4005867	20.0	3.37	0.69	9.9	Pokkali
*q*FETOX-7	7	id7000519	3.7	3.87	1.11	11.7	Pokkali
*q*FETOX-12	12	id12010050	27.6	3.56	0.96	10.6	Pokkali

^a^Closely linked markers are assumed as the same QTL.

^b^Marker associated with QTL.

^c^Physical position of markers on chromosomes.

^d^F-statistical analysis indicates association between markers and trait.

^e^Positive/negative values indicate IR29/Pokkali can increase trait values.

^f^Proportion of phenotypic variance explained.

^g^Tolerance allele provided by parental line.

### Fe uptake analysis in contrasting lines in IR29/Pokkali population

**Figure 2:**
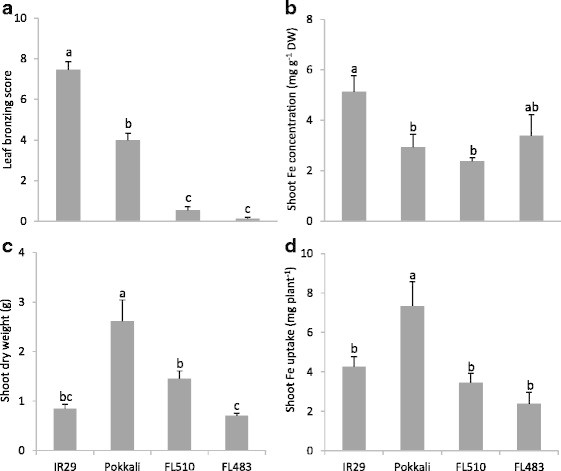
**Phenotypic traits of contrasting lines in IR29/Pokkali population under 1,000 mg L**^**−1**^**Fe**^**2+**^**for 5 days were determined: (a) leaf bronzing score; (b) Fe concentration in shoot; (c) shoot dry weight; (d) total Fe uptake in shoot.** Vertical bars represent standard errors of means (n = 4). Different letters above the data points indicate significant differences between genotypes by LSD-test (*p* < 0.05).

Based on the screening, FL510 and FL483, which showed significantly lower leaf bronzing scores (Figure 2a) than both parents, were chosen for Fe uptake analysis. FL510 carried the tolerance alleles of all QTLs detected in IR29/Pokklai population, while FL483 only carried tolerance alleles at *q*FETOX-1-1 and *q*FETOX-1-2 (Table 1). Compared to IR29, the shoot Fe concentration was significantly lower in Pokkali and FL510, while FL483 did not differ significantly from any other genotypes (Figure 2b). Lower Fe concentrations in Pokkali despite higher absolute Fe uptake (Figure 2d) may have partly occurred due to higher biomass (Figure 2c) leading to a ‘dilution’ effect. However, FL510 had even lower Fe concentration than Pokkali (Figure 2b), despite a significantly lower biomass than Pokkali (Figure 2c), suggesting that dilution was not the dominant factor leading to low Fe concentrations in FL510. FL483 did not differ significantly from IR29 in shoot Fe concentration, dry weight, or shoot Fe uptake, suggesting that it was tolerant due to a shoot-based mechanism.

### Root oxidizing power and microscopy test

**Figure 3:**
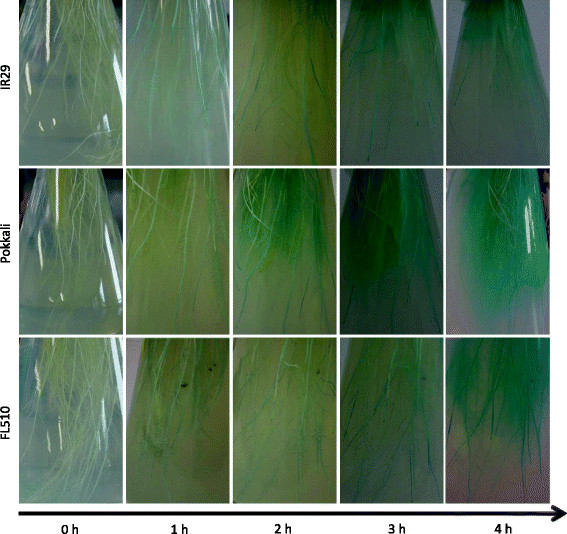
**Time course of root oxidizing power of contrasting lines in IR29/Pokkali population were indicated by color change in Methylene-blue agar solution.** Representative photos of 4 replicates per genotype are shown. Horizontal axis represents the time of duration (0–4 hours). Blue color indicates the site of oxygen release from roots.

**Figure 4:**
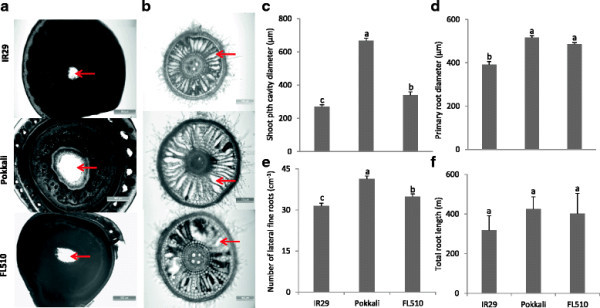
**Architectural traits related to rhizosphere aeration of contrasting lines in IR29/Pokkali population.** Representative images of the pith cavity in shoots **(a)** and primary roots **(b)** in the three lines were documented after 5 days of Fe stress. Red arrows indicate the aerenchyma. The diameter of pith cavity **(c)** and primary roots **(d)** were determined and vertical bars indicate standard errors of means (n = 4). The numbers of lateral fine roots within 1 cm distance from primary roots were counted **(e)**, and vertical bars represent standard errors of means (n = 20). The total length of roots **(f)** was determined by root scanning; vertical bars indicate standard errors of means of n = 3 (IR29 and FL510) or n = 4 (Pokkali). Different letters indicate significant differences between genotypes by LSD-test (*p* < 0.05).

To investigate the physiological basis of low shoot Fe concentration in FL510, the oxidizing power of roots as a main factor in excluding Fe in the rhizosphere was investigated. Oxidation of the rhizosphere as indicated by a color change of the Methylene-blue indicator proceeded at a faster pace in Pokkali than in IR29, while FL510 was intermediate (Figure 3). The color change was first observed around the root tips and lateral fine roots, suggesting that these sites are important for root oxygen release. To investigate the mechanisms associated with differences in root oxidizing power among IR29, Pokkali and FL510, plant shoots and roots were tested for architectural traits (Figure 4a, b). The results showed that at the same growth stage, Pokkali and FL510 had aerenchyma with a larger diameter in the pith cavity than IR29 (Figure 4c). The degree of aerenchyma differentiation in primary roots among the lines was not obviously different (Figure 4b). However, the difference of primary root diameters was significant (*p* < 0.05) between contrasting lines (Figure 4d). The density of lateral fine roots was measured by counting their number within 1 cm of the primary roots. As shown in Figure 4e, the lateral fine roots of Pokkali and FL510 were denser than of IR29. Even though the trend of total root length was consistent with density of lateral fine roots, the difference among these lines was not significant (Figure 4f).

### QTL analysis in Nipponbare/Kasalath population

In the Nipponbare/Kasalath population (Table 2), one putative QTL *q*FETOX-1-3 was mapped in the marker interval of C742-C86 by using the trait of leaf bronzing score after 2-days-treatment on chromosome 1. It explained 18.6% of the phenotypic variation. On chromosome 3, *q*FETOX-3 was mapped between C136 and R250 for both leaf bronzing score after 2 and 5 days of treatment. On chromosome 8, *q*FETOX-8 with an LOD value of 3.96 was detected, and it explained 17% of the phenotypic variation. For the QTLs on chromosome 1 and 8, tolerance alleles were provided by Nipponbare, whereas Kasalath contributed the tolerance allele to the QTL on chromosome 3.

**Table 2:** QTLs associated with leaf bronzing scores in Nipponbare/Kasalath population by composite interval mapping

**QTL**	**Trait**^**a**^	**Chr**^**b**^	**Marker interval**^**c**^	**Support interval (cM)**^**d**^	**LOD value**	**R**^**2**^**(%)**^**e**^	**Additive effect**^**f**^	**Tolerance allele**^**g**^	**CSSLs**^**h**^
*q*FETOX-1-3	LBS2	1	C742-C86	16.7-26.9	4.38	18. 6	−0.53	Nipponbare	SL54
*q*FETOX-3	LBS2	3	C136-R250	23.7-37.7	2.63	11. 6	0.44	Kasalath	SL15
	LBS5	3	C136-R250	23.7-37.7	3.71	16.0	0.52	Kasalath	SL15
*q*FETOX-8	LBS5	8	R727-C166	44.0-70.7	3.96	17.0	−0.51	Nipponbare	SL38

^a^LBS2: leaf bronzing score after 2 days of treatment, LBS5: leaf bronzing score after 5 days of treatment.

^b^Chromosomes number on which QTLs were detected.

^c^QTL was located between the markers.

^d^The position of marker associated with QTL on linkage map.

^e^Proportion of phenotypic variance explained.

^f^Positive/negative values indicate Nipponbare/Kasalath can increase trait values.

^g^Tolerance allele provided by parental line.

^h^Chromosome segment substitution lines carrying Kasalath genetic inserts related to specific QTLs.

### Confirmation of QTL using chromosome segment substitution lines

**Figure 5:**
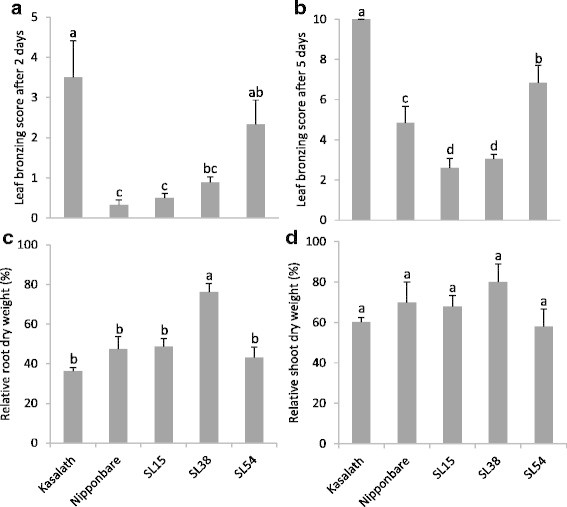
**Phenotypic traits of chromosome segment substitution lines SL15 and SL54 and two parents tested in 1,000 mg L**^**−1**^**Fe**^**2+**^**for 5 days. (a)** Leaf bronzing score after 2-days-treatment, **(b)** leaf bronzing score after 5-days-treatment, **(c)** relative root dry weight and **(d)** relative shoot dry weight were determined. Vertical bars indicate standard errors of means (n = 4). Different letters indicate significant differences between genotypes by LSD-test (*p* < 0.05).

To confirm the QTLs detected in Nipponbare/Kasalath population, chromosome segment substitution lines (SL) carrying Kasalath inserts in Nipponbare genetic background were used. SL54 carried the intolerance allele at the position of *q*FETOX-1-3, SL15 carried the tolerance allele at the location of *q*FETOX-3 and SL38 carried the intolerance allele at the position of *q*FETOX-8 (Additional file 1: Figure S1). SL54 showed significantly higher (*p* < 0.01) leaf bronzing scores after 2 and 5 days than Nipponbare (Figure 5a, b) in accordance with the expected QTL effect (Table 2). For *q*FETOX-3, SL15 showed significantly lower bronzing scores after five days (*p* < 0.01) than Nipponbare in accordance with the expected effect (Table 2). However, no significant differences in relative root and shoot biomass were found between two SLs and Nipponbare (Figure 5c, d). Overall, the effect of the two QTLs, *q*FETOX-1-3 and *q*FETOX-3 were confirmed. For *q*FETOX-8, the associated substitution line SL38 showed similar leaf bronzing score after 2-days-treatment as Nipponbare (Figure 5a). However, bronzing scores (5 days) of SL38 was lower than in Nipponbare which was contrary to the expected QTL effect (Figure 5b). Also for the root biomass, SL38 showed significantly higher values than Nipponbare, while no significant differences were observed for shoot biomass (Figure 5c). Due to inconclusive data, the effect of *q*FETOX-8 was not confirmed.

### Analysis of shoot Fe uptake in SLs in Nipponbare/Kasalath population

**Figure 6:**
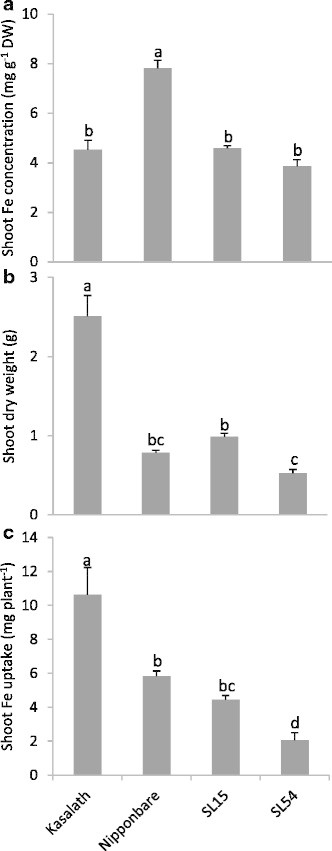
**Shoot Fe concentration, dry weight, and Fe uptake and dry weight in SL15 and SL54 and their two parents after treatment with 1,000 mg L**^**−1**^**Fe**^**2+**^**for 5 days. (a)** Shoot Fe concentration, **(b)** shoot dry weight, and **(c)** total Fe uptake were determined. Vertical bars indicate standard errors of means (n = 4). Different letters indicate significant differences between genotypes by LSD-test (*p* < 0.05).

Fe concentration was analyzed in those SLs carrying confirmed QTL along with their parents to gain some insight into the associated mechanism. Kasalath was extremely sensitive to Fe stress despite significantly lower Fe concentration than Nipponbare (Figure 6a). The low concentration may be a result of high biomass (Figure 6b), leading to a ‘dilution effect’. Similarly, both SLs had similar Fe concentrations, which were significantly lower than in Nipponbare, but they did not differ significantly from Nipponbare in terms of biomass (Figure 6b). This suggests that *q*FETOX-1-3 (SL54) conferred Fe sensitivity via a shoot based mechanism, while *q*FETOX-3 (SL15) conferred Fe tolerance via Fe exclusion.

## Discussion

Leaf bronzing symptoms were often used as a phenotypic trait in studies on abiotic stresses. They can quickly be scored for a large number of plants and are associated with many abiotic stresses in rice, *e.g.* Zn deficiency and ozone stress (Frei et al. [2010]; Höller et al. [2014]). Under Fe-toxic conditions, leaf bronzing was strongly correlated with yield loss. It was estimated that each visual symptom score increment is associated with a yield loss of approximately 400 kg ha^−1^ (Audebert and Fofana [2009]). Thus, we considered leaf bronzing score as a relevant trait for the screening of tolerance to Fe toxic conditions.

**Figure 7:**
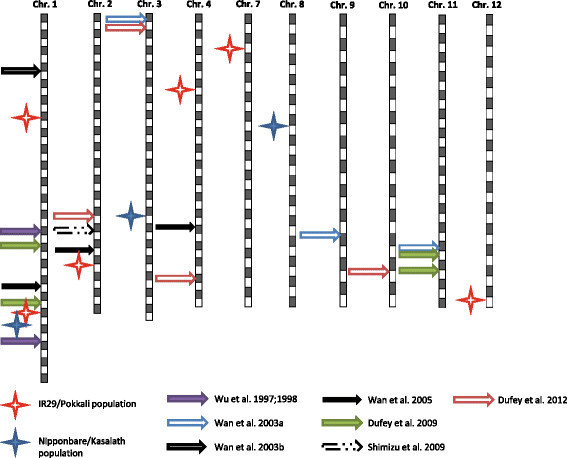
**Co-localization analysis of QTL reported in this study with previously reported QTLs for leaf bronzing under Fe toxic condition sin rice.** QTLs were located on chromosomes based on the physical positions of flanking markers. One quadrate (in grey or white) represents 1 Mb. Stars represent the QTLs mapped in this study and arrows represent the QTLs from other previous reports.

Contradictory rankings of tolerance were observed in many studies due to different screening conditions and different development stages at which plants were tested. Our study was targeting the intensive pulse stress at the early vegetative stage that typically occurs during rainfall events in a tropical inland valley landscape. We simulated this Fe pulse in nutrient solution maintained at low redox potential by gaseous N_2_ percolation (Engel et al. [2012]). Genetic analyses of tolerance to Fe toxicity have been reported in several previous studies, although most of them used different screening conditions, and they focused on chronic rather than pulse stresses. Putative QTLs detected in our study were compared with previous reports based on the physical positions of associated markers in the Nipponbare genome (International Rice Genome Sequencing Project, http://rgp.dna.affrc.go.jp/IRGSP/). Based on leaf bronzing scores and other physiological traits, QTLs associated with tolerance to Fe toxicity were mapped on the whole genome except for chromosome 5 and 6 (Figure 7). On chromosome 1, *q*FETOX-1-2 in IR29/Pokkali population and *q*FETOX-1-3 in Nipponbare/Kasalath population co-localized with the QTLs detected by Dufey et al. ([2009,2012]) and Wu et al. ([1998]). Another QTL in the IR29/Pokkali population, *q*FETOX-2 was mapped close to a QTL reported by Wan et al. ([2005]). For other QTLs in our study, no co-localization was found with previous reports, except for *q*FETOX-7 which was mapped close to one QTL for physiological trait by Wu et al. ([1998]). The fact that there is multitude of rather small effect QTLs underlines the concept of multiple tolerance mechanisms involved in different types of Fe toxicity, for example chronic versus pulse stress. This view is also supported by a transcriptomic study in which gene regulations under short term Fe stress were substantially different from chronic Fe stress in rice (Quinet et al. [2012]).

To shed some light on mechanisms involved in tolerance to Fe pulse stress, we investigated Fe uptake and root morphological properties associated with Fe oxidation. Oxidation of Fe^2+^ at root surface typically leads to the formation of Fe plaque that has also been proposed to act as a barrier or buffer to reduce the uptake of toxic elements (e.g. Al, Cd, As) into plant tissues (Liu et al. [2004]; Liu et al. [2010]; Chen et al. [2006]). Root oxidizing power as a direct cause of Fe^2+^ oxidation is assumed to be composed of two distinct processes: root oxygen release and enzymatic oxidation (Ando et al. [1983]). The aerenchyma provides a low resistance pathway for internal oxygen movement within the roots (Colmer [2002]). Our results suggest that larger diameter of the pith cavity in shoot that favors the oxygen transport from shoot to root, along with larger primary root diameter, which could increase the absolute volume of aerenchyma, together with higher density of lateral fine roots was effective in increasing the root oxidation power and thus increasing Fe exclusion ability in Pokkali and FL510. Similarly, a QTL (*q*FETOX-3) associated with Fe exclusion was identified in the Nipponbare/Kasalath population, which tolerance allele was carried by SL15. The same line, SL15 was previously shown to be quite sensitive to ozone stress (Frei et al. [2008]), indicating that it was not tolerant to oxidative stress in the shoot, but that tolerance in this line was clearly associated with Fe exclusion. According to the Rice Annotation Project Database (RAP-DB, http://rapdb.dna.affrc.go.jp/index.html), one Fe regulated transporter gene, *OsIRT1* (Os03g0667500) is localized within the *q*FETOX-3 region. Rice plants employ *OsIRT1* for acquisition of Fe^2+^ (Ishimaru et al. [2006]) indicating that polymorphisms in this gene could be responsible for the Fe exclusion mechanism.

Our study also revealed several QTLs apparently associated with shoot based mechanisms, which may be composed of two components: Fe compartmentation and ROS detoxification. Higher amount of Fe in low-molecular-mass fractions (<3 kDa) was found in the leaf extracts of sensitive genotype suggesting Fe stored in high-molecular-mass fractions as a possible mechanism for shoot based tolerance (Stein et al. [2009]). Ferritin as Fe storage protein may play an important role in Fe compartmentation as the higher accumulation of ferritin mRNA and protein was observed in tolerant genotype (da Silveira et al. [2009]). Antioxidant enzyme activities and antioxidants are important for ROS detoxicification. It is reported that ascorbate peroxidase, glutathione reductase and peroxidase activities were increased in rice leaf by excess Fe (Fang et al. [2001]; Fang and Kao [2000]; Stein et al. [2009]). External application of mannitol and reduced glutathione could scavenge the damage from Fe toxicity (Fang et al. [2001]). However, to our knowledge, there is no direct evidence of constitutively high content of antioxidants or antioxidant enzymes activities leading to elevated tolerance to Fe toxicity. Our study suggests that shoot based mechanisms may be effective in protecting plants from Fe pulse stress. In particular, FL483 carrying the favorable allele of the *q*FETOX-1-2 showed markedly high tolerance with similar shoot Fe concentration with intolerant parent IR29. In the Nipponbare/Kasalath population, SL54 carried the intolerant allele of *q*FETOX-1-3 (which was located near *q*FETOX-1-2) and showed high shoot sensitivity, suggesting that this segment on chromosome 1 was related to shoot-based tolerance. According to RAP-DB, two similar genes Os01g0878800 and Os01g0878900 are localized within the two QTLs at 38,466 kb on chromosome 1 (Figure 7). Based on the gene ontology database of the European Bioinformatics Institute (https://www.ebi.ac.uk/QuickGO/), the molecular functions of these two genes were described as Fe^2+^ binding (GO: 0008198). Another interesting gene, Os01g0837800 (similar to metal tolerance gene, from RAP-DB) is localized at 35,917 kb on chromosome 1 which is close to *q*FETOX-1-2. Further investigations are warranted to elucidate the possible mechanisms related to these loci.

## Conclusion

We identified a number of QTLs related to tolerance of Fe pulse stresses in the vegetative growth stage of rice. Some of these QTL co-localized with previously reported QTL that were mapped under more chronic Fe stress, suggesting that they were associated with ‘universal’ defense mechanisms. However, the majority of QTLs had rather small effects and was distributed throughout the genome, confirming the complexity of the genetics behind adaptation to varying Fe toxic conditions. Further, QTLs were associated with either exclusion or inclusion mechanisms of Fe tolerance. We suggest that Fe exclusion via oxidation at the root surface is an important adaptive trait under Fe pulse stress. The trait appears to be favored by root architecture and can be genetically dissected within the IR29/Pokkali mapping population. Pyramiding this trait with further shoot based adaptive traits may be effective in the breeding for Fe toxicity tolerance.

## Methods

### Plant material

Two pairs of parents from mapping populations were screened initially for tolerance to an Fe pulse stress at the vegetative growth stage: IR29/Pokkali, and Nipponbare/Kasalath. Based on contrasting tolerance response patterns, these populations were selected to identify tolerance QTL for Fe pulse stresses. An F_8_ recombinant inbred (RIL) population consisting of 121 lines was derived from a cross of two *indica* varieties IR29 (intolerant) and Pokkali (tolerant) (Gregorio [1997]). Pokkali was characterized as tolerant to intensive pulse stresses during the vegetative growth stage due to its Fe exclusion capacity (Engel et al. [2012]). Another population consisted of 98 BC_1_F_5_ lines derived from a backcross of Nipponbare/Kasalath//Nipponbare by the single-seed descent method (Taguchi-Shiobara et al. [1997]). Nipponbare was characterized as a moderately tolerant *japonica* variety (Engel et al. [2012]), whereas Kasalath was a highly sensitive *Aus* variety. Further chromosome segment substitution lines (SL) carrying small Kasalath inserts in a Nipponbare genetic background were provided by the Rice Genome Resource Center of NIAS, Japan.

### Hydroponic culture and screening

Experiments were conducted in a glasshouse with the day/night temperature set as 30/25°C, and natural light with supplementary lighting to ensure a minimum photosynthetically active radiation (PAR) of 400 μmol m^−2^ sec^−1^. Rice seeds were soaked in demineralized water and germinated at 30°C in the dark for 72 hours. Subsequently, germinating seeds were floated in 70.5 mg L^−1^ CaCl_2_ and 1.625 mg L^−1^ FeCl_3_ solution in light for another 5 days. Homogenous seedlings were selected and transplanted into 40 L tanks filled with half-strength nutrient solution (Yoshida et al. [1976]). In all experiments, at least four replicate plants per genotype were used, except for the QTL mapping of the IR29/Pokkali population, where the number of available containers could only accommodate 3 replicates per line. After 10 days, nutrient solutions were changed to full-strength with the following composition: : 40 mg L^−1^ N (as NH_4_NO_3_), 10 mg L^−1^ P (as NaH_2_PO_4_ · 2H_2_O), 40 mg L^−1^ K (as K_2_SO_4_), 40 mg L^−1^ Ca (as CaCl_2_), 40 mg L^−1^ Mg (as MgSO_4_ · 7H_2_O), 0.5 mg L^−1^ Mn (as MnCl_2_ · 4H_2_O), 0.05 mg L^−1^ Mo (as (NH_4_)_6_ · MO_7_O_24_ · 4H_2_O), 0.2 mg L^−1^ B (as H_3_BO_3_), 0.01 mg L^−1^ Zn (as ZnSO_4_ · 7H_2_O), 0.01 mg L^−1^ Cu (as CuSO_4_ 5H_2_O), 2 mg L^−1^ Fe (as FeCl_3_x6H_2_O with 14.9 mg L^−1^ citric acid monohydrate). During the whole growing period, the pH value was adjusted to 5.5 every other day and solutions were completely exchanged every 10 days. The roots of plants grown in the same container were separated using PVC tubes fixed underneath a perforated covering plate.

After 5 weeks of growth, half of the rice plants were exposed to a pulse stress of 1000 mg L^−1^ (=17.86 mM) Fe^2+^ (as FeSO_4_ · 7H_2_O) for 5 days. Maintaining a low redox potential (Eh) in solution prevents Fe^2+^ from being re-oxidized and reportedly accelerates toxicity symptom expression (Wang et al. [2008]). Therefore, N_2_ gas was percolated into the culture solutions for 15 minutes every 2 hours to remove dissolved oxygen to keep the solution at low redox potential. Leaf bronzing scores were measured on the three youngest fully expanded leaves of the main tiller. Leaf bronzing score which indicates the severity of Fe toxicity range from 0 (healthy leaf without symptom) to 10 (dead leaf). Representative photos of different leaf bronzing scores were shown in Additional file 2: Figure S2 (adjusted from Wissuwa et al. [2006]). This trait was measured after two days of Fe treatment, representing an initial response to excess Fe, and after five days, when a more pronounced genotypic differentiation was observed. Plant materials were harvested for further investigation. Shoot dry weight and root dry weight were measured. The reduction of shoot and root growth were calculated as: Relative Shoot Dry Weight = (shoot dry weight in treatment)/(shoot dry weight in control) × 100%; Relative Root Dry Weight = (root dry weight in treatment)/(root dry weight in control) × 100%.

### QTL mapping and statistical analysis

For the IR29/Pokkali population, a physical map consisted of 173 SNP and 83 SSR markers covering all 12 rice chromosomes based on the physical locations. QTL analysis was performed by single marker regression analysis (Kearsey and Hyne [1994]) with Qgene 4.3 (Joehanes and Nelson [2008]). A -log *p*(F) ≥ 3.0 was taken as the threshold for the detection of putative QTL. For Nipponbare/Kasalath population, the genetic linkage map was composed of 245 RFLP markers (Lin et al. [1998]). QTLs were analyzed by the composite interval mapping method (Zeng [1994]) with Qgene 4.3. LOD value ≥ 2.5 was used as the threshold of for the declaration of putative QTL. Statistical software SPSS was applied for analysis of variance (ANOVA, IBM SPSS Statistics 21). Pair-wise genotypic differences were determined by post-hoc comparison using the LSD-Test and Tukey adjustment was used for multiple comparison of means if appropriate.

### Physiological analysis of contrasting lines

Plant shoots of contrasting lines were oven-dried at 60°C until the weight was constant and ground to a fine powder. Fe concentrations in shoots were determined after digesting 250 mg of dry samples with 4 ml 65% HNO_3_ at 180°C for 8 hours followed by dilution to 25 ml and filteration. Standard and sample solutions were measured using atomic absorption spectroscopy (AAS, Perkin-ELMER 1100B, Überlingen, Germany).

The redox indicator methylene blue was applied to detect the root oxidizing power (Kotula [2009]). Solution containing 0.75% agar was boiled and then cooled down to 60°C with continuous percolation of N_2_ gas to completely remove the dissolved oxygen. Methylene blue was added into agar solution at a concentration of 2 mg L^−1^. The blue mixture containing the oxidized dye was furtherly cooled down to 35°C. Then, 0.75 g L^−1^ sodium dithionite (Na_2_S_2_O_4_) was added to reduce methylene blue and the solution turned to colorless. The roots of 4 representative plants of each line were carefully placed in 500 ml Erlenmeyer flasks. Gaseous N_2_ was applied to remove air from the flasks. Colorless solution containing reduced methylene blue was poured into the flasks to submerge the whole root system. The open surface of flasks was immediately covered with a plastic wrap to avoid air diffusion. Flasks were wrapped with aluminum foil to keep the roots in the dark. The plants were placed in a greenhouse at 30°C for 4 hours. Photographs were taken at every hour to record the color changes in rhizosphere due to the root oxdizing power.

To observe the aerenchyma formation in root and shoot, 4 plants of each line were selected. Primary roots were detached at 2 mm distance from shoot base and sliced vertically using a razor blade. The main tiller of each line was also sliced at the shoot base. The slices were observed using a light microscope (Leica DFC425, Heerbrugg, Germany) and photographs were taken to show the aerenchyma formation and measure the diameter of primary root and pith cavity in shoot. Root systems of contrasting lines were scanned (EPSON, EU-88, Seiko Epson Corp. Japan) and lateral fine roots within 1 cm on primary roots were counted at 10 different sites of each plant. Root total length of each plant was measured by the software XLRhizo 2012b (Regent Instruments Inc. Canada).

## Abbreviations

LOD: Logarithm of odds: 

QTL: Quantitative trait locus: 

RIL: Recombinant inbred line: 

ROS: Reactive oxygen species: 

SL: Chromosome segment substitution line: 

## Competing interests

The authors declare that they have no competing interests.

## Authors’ contributions

MF, MB, and LBW conceived the project. GG provided the IR29/Pokkali population and the 173 SNP and 83 SSR markers. LBW and MYS and EM performed the mapping and physiological analysis of the. LBW and MF analyzed the data and drafted the manuscript. All authors read and approved the final manuscript.

## Additional files

## Supplementary Material

Additional file 1: Figure S1 Schematic representation of the genotypes of chromosome segment substitution lines (SL), SL15, SL38 and SL54. Black and white bars represent Kasalath and Nipponbare segments, respectively. Grey bars indicate heterozygous segment. Approximate positions of three putative QTLs detected in Nipponbare/Kasalath population were shown. The effect of *q*FETOX-1-3 was to decrease leaf bronzing score and *q*FETOX-3 and *q*FETOX-8 can increase the leaf bronzing score. Click here for file

Additional file 2: Figure S2 Representative photos of leaf bronzing scores ranging from 0 (healthy leaf) to 10 (dead leaf). Click here for file
