# Supplementary figures and images for: Genetic and physiological analysis of tolerance to acute iron toxicity in rice
Source: Rice (N Y). 2014 May 30;7(1):8. doi: 10.1186/s12284-014-0008-3 (PMC4052628; doi:10.1186/s12284-014-0008-3)

## Slide 1
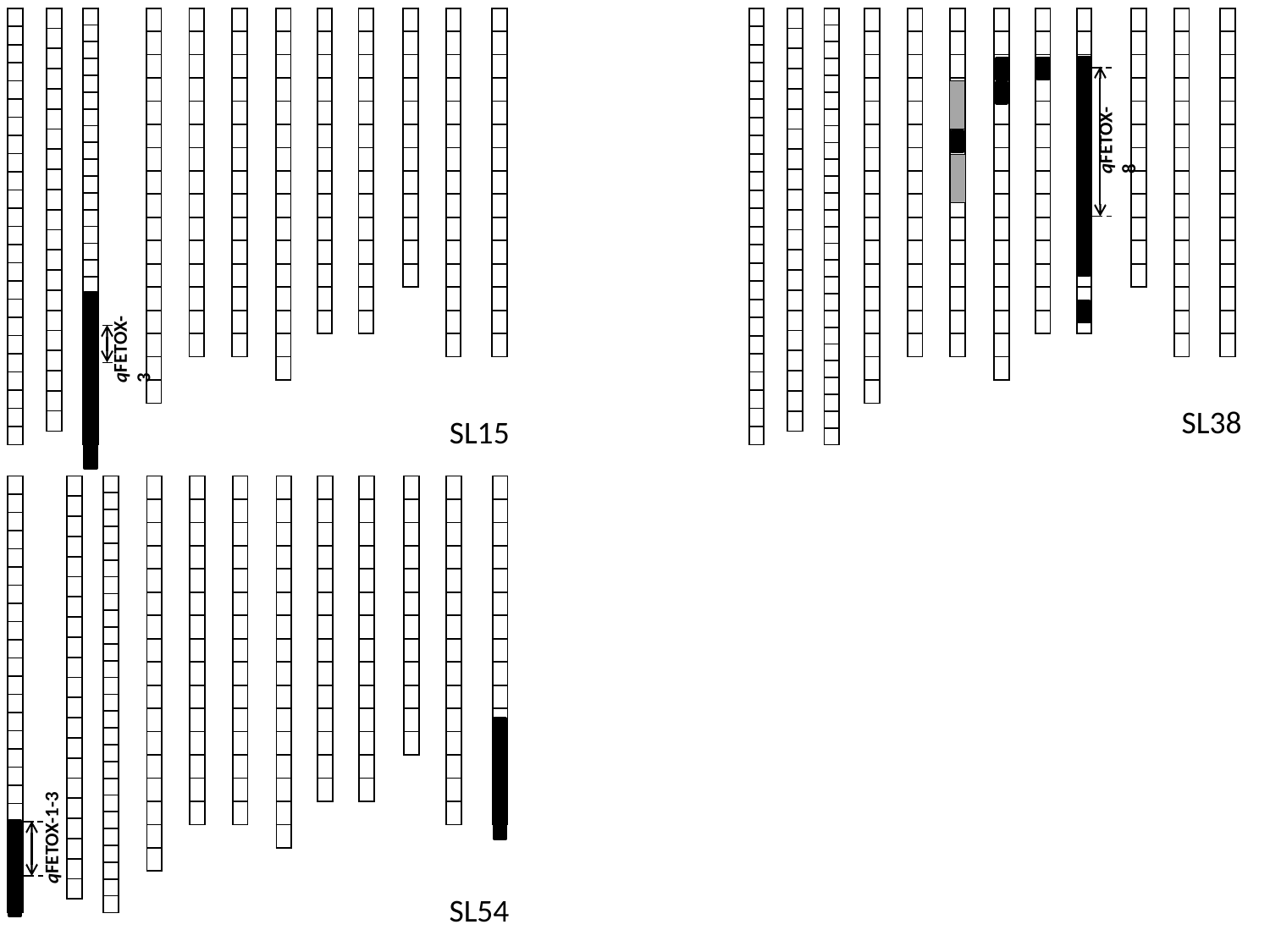

| |
| --- |
| |
| |
| |
| |
| |
| |
| |
| |
| |
| |
| |
| |
| |
| |
| |
| |
| |
| |
| |
| |
| |
| |
| |
| |
| --- |
| |
| |
| |
| |
| |
| |
| |
| |
| |
| |
| |
| |
| |
| |
| |
| |
| |
| |
| |
| |
| |
| --- |
| |
| |
| |
| |
| |
| |
| |
| |
| |
| |
| |
| |
| |
| |
| |
| |
| |
| |
| |
| |
| |
| |
| |
| |
| |
| |
| --- |
| |
| |
| |
| |
| |
| |
| |
| |
| |
| |
| |
| |
| |
| |
| |
| |
| |
| --- |
| |
| |
| |
| |
| |
| |
| |
| |
| |
| |
| |
| |
| |
| |
| |
| --- |
| |
| |
| |
| |
| |
| |
| |
| |
| |
| |
| |
| |
| |
| |
| |
| --- |
| |
| |
| |
| |
| |
| |
| |
| |
| |
| |
| |
| |
| |
| |
| |
| |
| --- |
| |
| |
| |
| |
| |
| |
| |
| |
| |
| |
| |
| |
| |
| |
| --- |
| |
| |
| |
| |
| |
| |
| |
| |
| |
| |
| |
| |
| |
| |
| --- |
| |
| |
| |
| |
| |
| |
| |
| |
| |
| |
| |
| |
| --- |
| |
| |
| |
| |
| |
| |
| |
| |
| |
| |
| |
| |
| |
| |
| |
| --- |
| |
| |
| |
| |
| |
| |
| |
| |
| |
| |
| |
| |
| |
| |
| |
| --- |
| |
| |
| |
| |
| |
| |
| |
| |
| |
| |
| |
| |
| |
| |
| |
| |
| |
| |
| |
| |
| |
| |
| |
| |
| --- |
| |
| |
| |
| |
| |
| |
| |
| |
| |
| |
| |
| |
| |
| |
| |
| |
| |
| |
| |
| |
| |
| --- |
| |
| |
| |
| |
| |
| |
| |
| |
| |
| |
| |
| |
| |
| |
| |
| |
| |
| |
| |
| |
| |
| |
| |
| |
| |
| |
| --- |
| |
| |
| |
| |
| |
| |
| |
| |
| |
| |
| |
| |
| |
| |
| |
| |
| |
| --- |
| |
| |
| |
| |
| |
| |
| |
| |
| |
| |
| |
| |
| |
| |
| |
| --- |
| |
| |
| |
| |
| |
| |
| |
| |
| |
| |
| |
| |
| |
| |
| |
| --- |
| |
| |
| |
| |
| |
| |
| |
| |
| |
| |
| |
| |
| |
| |
| |
| |
| --- |
| |
| |
| |
| |
| |
| |
| |
| |
| |
| |
| |
| |
| |
| |
| --- |
| |
| |
| |
| |
| |
| |
| |
| |
| |
| |
| |
| |
| |
| |
| --- |
| |
| |
| |
| |
| |
| |
| |
| |
| |
| |
| |
| |
| --- |
| |
| |
| |
| |
| |
| |
| |
| |
| |
| |
| |
| |
| |
| |
| |
| --- |
| |
| |
| |
| |
| |
| |
| |
| |
| |
| |
| |
| |
| |
| |
qFETOX-8
qFETOX-3
SL38
SL15
| |
| --- |
| |
| |
| |
| |
| |
| |
| |
| |
| |
| |
| |
| |
| |
| |
| |
| |
| |
| |
| |
| |
| |
| |
| |
| |
| --- |
| |
| |
| |
| |
| |
| |
| |
| |
| |
| |
| |
| |
| |
| |
| |
| |
| |
| |
| |
| |
| |
| --- |
| |
| |
| |
| |
| |
| |
| |
| |
| |
| |
| |
| |
| |
| |
| |
| |
| |
| |
| |
| |
| |
| |
| |
| |
| |
| |
| --- |
| |
| |
| |
| |
| |
| |
| |
| |
| |
| |
| |
| |
| |
| |
| |
| |
| |
| --- |
| |
| |
| |
| |
| |
| |
| |
| |
| |
| |
| |
| |
| |
| |
| |
| --- |
| |
| |
| |
| |
| |
| |
| |
| |
| |
| |
| |
| |
| |
| |
| |
| --- |
| |
| |
| |
| |
| |
| |
| |
| |
| |
| |
| |
| |
| |
| |
| |
| |
| --- |
| |
| |
| |
| |
| |
| |
| |
| |
| |
| |
| |
| |
| |
| |
| --- |
| |
| |
| |
| |
| |
| |
| |
| |
| |
| |
| |
| |
| |
| |
| --- |
| |
| |
| |
| |
| |
| |
| |
| |
| |
| |
| |
| |
| --- |
| |
| |
| |
| |
| |
| |
| |
| |
| |
| |
| |
| |
| |
| |
| |
| --- |
| |
| |
| |
| |
| |
| |
| |
| |
| |
| |
| |
| |
| |
| |
qFETOX-1-3
SL54

Supplement: Additional file 1: Figure S1 — Schematic representation of the genotypes of chromosome segment substitution lines (SL), SL15, SL38 and SL54. Black and white bars represent Kasalath and Nipponbare segments, respectively. Grey bars indicate heterozygous segment. Approximate positions of three putative QTLs detected in Nipponbare/Kasalath population were shown. The effect of qFETOX-1-3 was to decrease leaf bronzing score and qFETOX-3 and qFETOX-8 can increase the leaf bronzing score. [file s12284-014-0008-3-S1.pptx]

## Slide 1
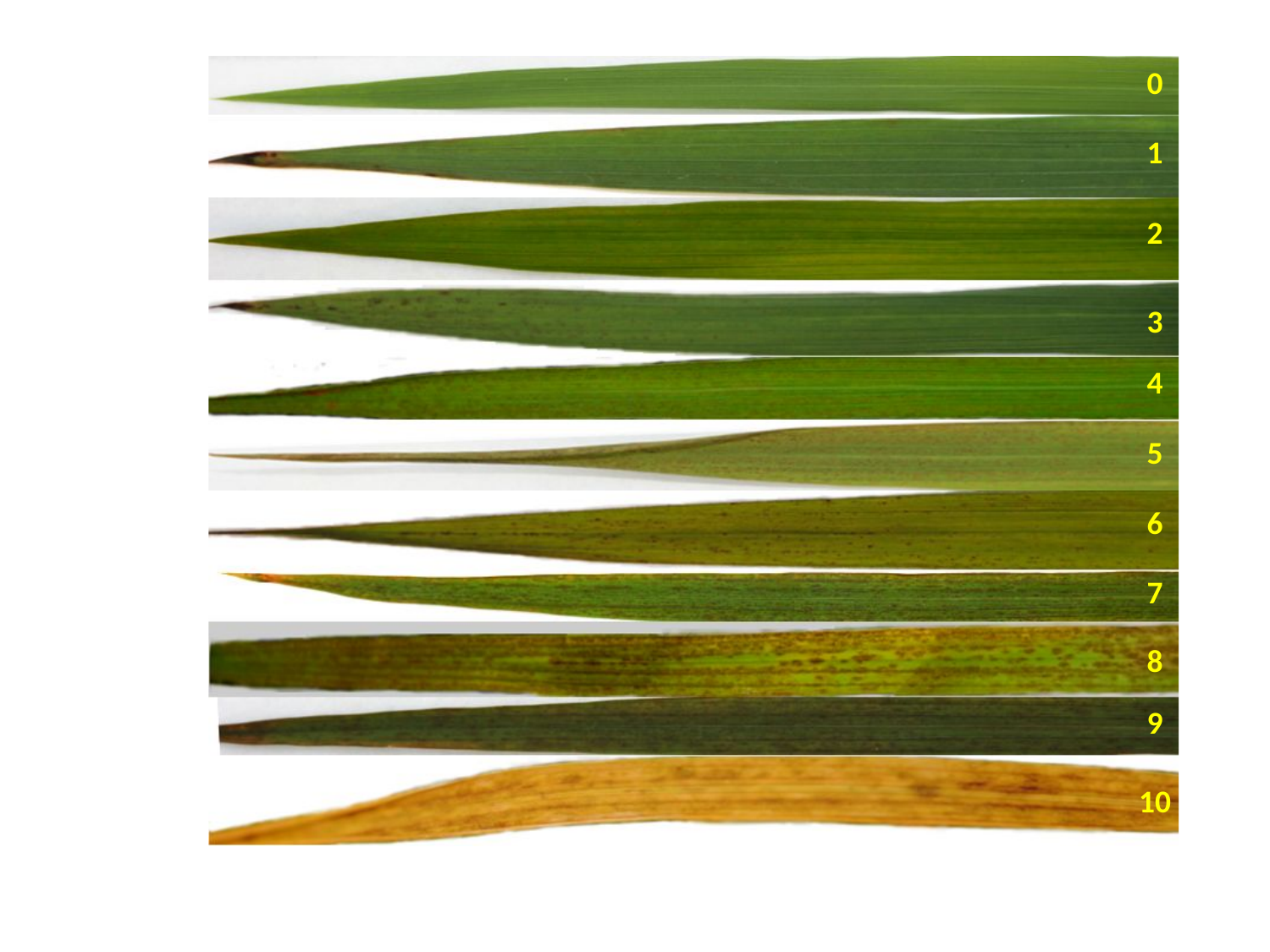

0
1
2
3
4
5
6
7
8
9
10

Supplement: Additional file 2: Figure S2 — Representative photos of leaf bronzing scores ranging from 0 (healthy leaf) to 10 (dead leaf). [file s12284-014-0008-3-S2.pptx]
